# Dapagliflozin in chronic kidney disease: cost-effectiveness beyond the DAPA-CKD trial

**DOI:** 10.1093/ckj/sfae025

**Published:** 2024-02-09

**Authors:** Phil McEwan, Jason A Davis, Peter D Gabb, David C Wheeler, Peter Rossing, Glenn M Chertow, Ricardo Correa-Rotter, Kouichi Tamura, Salvatore Barone, Juan Jose Garcia Sanchez

**Affiliations:** Health Economics and Outcomes Research Ltd, Cardiff, UK; Health Economics and Outcomes Research Ltd, Cardiff, UK; Health Economics and Outcomes Research Ltd, Cardiff, UK; Department of Renal Medicine, University College London, London, UK; Steno Diabetes Centre Copenhagen, Herlev, Denmark; Department of Clinical Medicine, University of Copenhagen, Copenhagen, Denmark; Departments of Medicine and Epidemiology and Population Health, Stanford University School of Medicine, Stanford, CA, USA; Department of Nephrology and Mineral Metabolism, National Medical Science and Nutrition Institute Salvador Zubiran, Mexico City, Mexico; Department of Medical Science and Cardiorenal Medicine, Yokohama City University Graduate School of Medicine, Yokohama, Japan; Global Medical Affairs, AstraZeneca, Gaithersburg, MD, USA; Health Economic and Payer Evidence, AstraZeneca, Cambridge, UK

**Keywords:** albuminuria, chronic kidney disease, cost-effectiveness, dapagliflozin, SGLT2 inhibitor

## Abstract

**Background:**

The Dapagliflozin and Prevention of Adverse Outcomes in CKD (DAPA-CKD) trial enrolled patients with estimated glomerular filtration rate 25–75 mL/min/1.73 m^2^ and urine albumin-to-creatinine ratio >200 mg/g. The Dapagliflozin Effect on CardiovascuLAR Events-Thrombolysis in Myocardial Infarction 58 (DECLARE-TIMI 58) trial enrolled patients with type 2 diabetes, a higher range of kidney function and no albuminuria criterion. The study objective was to estimate the cost-effectiveness of dapagliflozin in a broad chronic kidney disease population based on these two trials in the UK, Spain, Italy and Japan.

**Methods:**

We adapted a published Markov model based on the DAPA-CKD trial but to a broader population, irrespective of urine albumin-to-creatinine ratio, using patient-level data from the DAPA-CKD and DECLARE-TIMI 58 trials. We sourced cost and utility inputs from literature and the DAPA-CKD trial. The analysis considered healthcare system perspectives over a lifetime horizon.

**Results:**

Treatment with dapagliflozin was predicted to attenuate disease progression and extend projected life expectancy by 0.64 years (12.5 versus 11.9 years, undiscounted) in the UK, with similar estimates in other settings. Clinical benefits translated to mean quality-adjusted life year (QALY; discounted) gains between 0.45 and 0.68 years across countries. Incremental cost-effectiveness ratios in the UK, Spain, Italy and Japan ($10 676/QALY, $14 479/QALY, $7771/QALY and $13 723/QALY, respectively) were cost-effective at country-specific willingness-to-pay thresholds. Subgroup analyses suggest dapagliflozin is cost-effective irrespective of urinary albumin-to-creatine ratio and type 2 diabetes status.

**Conclusion:**

Treatment with dapagliflozin may be cost-effective for patients across a wider spectrum of estimated glomerular filtration rates and albuminuria than previously demonstrated, with or without type 2 diabetes, in the UK, Spanish, Italian and Japanese healthcare systems.

## Graphical Abstract

**Graphical Abstract:**
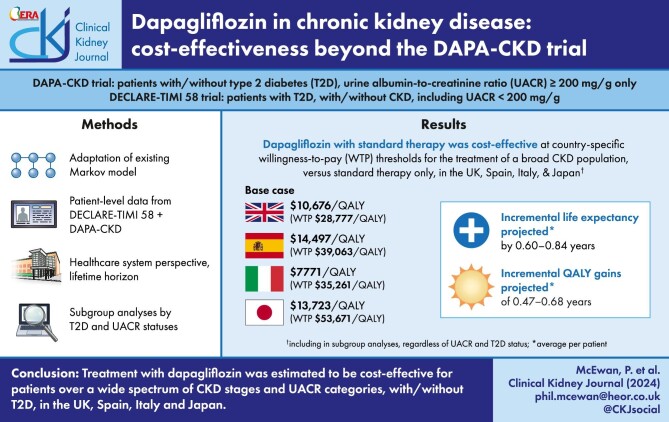

KEY LEARNING POINTS **What was known:**

- Chronic kidney disease (CKD) is associated with considerable burden to healthcare systems worldwide, particularly upon progression to kidney failure.
- The efficacy and safety of dapagliflozin in addition to standard therapy in patients with CKD and at least moderate degrees of albuminuria have been demonstrated in the DAPA-CKD trial, irrespective of type 2 diabetes status; the DECLARE-TIMI 58 trial demonstrated efficacy in patients with type 2 diabetes.

**This study adds:**

- By adapting a previously published model, this study assessed the cost-effectiveness of dapagliflozin in a broader CKD population using a subset of data from the DECLARE-TIMI 58 trial comprising patients who had CKD at baseline.
- Treatment with dapagliflozin was cost-effective, at country-specific willingness-to-pay thresholds; subgroup analyses suggested that dapagliflozin is expected to be cost-effective at lower levels of urinary albumin-to-creatine ratio and among patients with and without type 2 diabetes status.

**Potential impact:**

- This analysis addresses a data gap that currently exists in patients with CKD, suggesting that there may be value in treating patients with dapagliflozin across a wider spectrum of estimated glomerular filtration rates and albuminuria than previously demonstrated, with or without type 2 diabetes.

## INTRODUCTION

The prevalence of chronic kidney disease (CKD) is rising and is estimated to be 8%–16% worldwide [1], affecting an estimated 840 million people [2, 3]. CKD is usually a progressive condition, typically described by the level of estimated glomerular filtration rate (eGFR) and the presence and degree of albuminuria. Patients with reduced eGFR and/or albuminuria typically incur higher rates of hospitalization, outpatient visits, ambulance use and emergency room visits relative to patients with milder disease [4]. Accordingly, CKD has a detrimental effect on health-related quality of life (HRQoL) and yields a substantial burden to healthcare systems and society [5].

In addition to lifestyle changes, standard-of-care pharmacologic treatment options for CKD include angiotensin-converting enzyme inhibitors or angiotensin receptor blockers which aim to delay disease progression and prevent or ameliorate associated complications, yet many patients continue to progress toward advanced CKD.

Sodium-glucose co-transporter-2 (SGLT2) inhibitors are an established therapeutic option in patients with T2D. Trials designed to ensure the cardiovascular safety of SGLT2 inhibitors also demonstrated substantial benefits vis-à-vis cardiovascular outcomes distinct from their glucose lowering action. These and other studies showed protective effects on kidney function, including the potential to reduce the rate of eGFR decline and the risk of end-stage kidney disease (ESKD) in patients with type 2 diabetes (T2D) [6–12].

The efficacy and safety of dapagliflozin in addition to standard therapy in patients with CKD and albuminuria [eGFR 25–75 mL/min/1.73 m^2^; urinary albumin-to-creatine ratio (UACR) ≥200 mg/g] was investigated in the Dapagliflozin and Prevention of Adverse Outcomes in CKD (DAPA-CKD) trial [13]. The trial was ended prematurely due to overwhelming efficacy in patients with or without T2D. Subsequently, a cost-effectiveness analysis based on the DAPA-CKD trial demonstrated cost-effectiveness in multiple national healthcare systems [14]. While patients with CKD with lower levels of albuminuria or without albuminuria are generally at lower risk of adverse clinical outcomes, they may also gain benefit from treatment with an SGLT2 inhibitor [15, 16]. However, the potential value of treatment in these patients has not been studied.

The DAPA-CKD-like population typically have a higher per patient burden than the broad CKD population described in this analysis [17]. However, patients with lower UACR are more prevalent and therefore exert a higher burden at a population level on national healthcare systems [18]. Therefore, the largest benefit to patients and national healthcare systems could be attained by addressing the residual risk in patients outside of the DAPA-CKD population.

By adapting a previously published model driven by patient-level data from the DAPA-CKD trial, the objective of the present study is to assess the cost-effectiveness of dapagliflozin within the broader CKD population. Based on data from both the DAPA-CKD and Dapagliflozin Effect on CardiovascuLAR Events-Thrombolysis in Myocardial Infarction 58 (DECLARE-TIMI 58) trials, the model assesses dapagliflozin from national healthcare perspectives in the UK, Spain, Italy and Japan.

## MATERIALS AND METHODS

### Source data

A broad data set was derived by combining individual patient-level data from two placebo-controlled randomized controlled trials of dapagliflozin plus standard therapy. The DAPA-CKD trial included patients with eGFR 25–75 mL/min/1.73 m^2^ and UACR 200–5000 mg/g, with or without T2D. The DECLARE-TIMI 58 study included patients with T2D and estimated creatinine clearance ≥60 mL/min/1.73 m^2^ and no inclusion criteria based on UACR. Study designs, patient characteristics and outcomes have been published [8, 13, 19–23].

### The broad CKD population

We pooled individual patient data on patients with CKD from the two trials, including only the DECLARE-TIMI 58 subset of patients with eGFR <60 mL/min/1.73 m^2^ calculated by the CKD Epidemiology Collaboration equation [24, 25] and/or with albuminuria at baseline (>30 mg/g), referred to hereafter as the DECLARE_CKD_ population. Baseline patient characteristics of the pooled CKD population are provided in Table 1.

**Table 1:** Summary patient characteristics of pooled CKD populations from the DAPA-CKD and DECLARE TIMI 58 trials.

Characteristic	Pooled CKD (*N* = 10 273)
Demographic characteristics	
Age, years	63.3 (9.6)
Female	3365 (32.8)
Race	
White	6914 (67.3)
Asian	2367 (23.0)
Black	411 (4.0)
Other	581 (5.7)
Baseline body mass index, kg/m²	31.3 (6.3)
Current smoker	1482 (14.4)
Baseline systolic blood pressure, mmHg	137.3 (16.7)
Baseline diastolic blood pressure, mmHg	77.8 (9.8)
Haemoglobin, g/dL	13.4 (1.7)
Baseline serum potassium, mEq/L	4.5 (0.5)
Disease characteristics	
Baseline eGFR (mL/min per 1.73 m^2^)	
Mean (SD)	64.4 (24.7)
G1 (≥90)	2335 (22.7)
G2 (60–89)	2854 (27.8)
G3a (45–59)	2378 (23.1)
G3b (30–44)	2062 (20.1)
G4 (15–29)	640 (6.2)
G5 (<15)	4 (0.0)
Baseline UACR (mg/g)	
Median (IQR)	284 (62–932)
A1 (<30)	772 (7.5)
A2 (30–300)	4473 (43.5)
A3 (>300)	5028 (48.9)
Medical history	
Baseline T2D	8875 (86.4)
History of cardiovascular disease	3569 (34.7)
History of heart failure	1203 (11.7)
History of stroke	767 (7.5)
History of myocardial infarction	1760 (17.1)
ACE inhibitor or ARB	9170 (89.3)

Unless otherwise indicated, values for continuous variables are mean (SD); values for categorical variables represent counts (%).

ACE, angiotensin converting enzyme; ARB, angiotensin II receptor blocker; IQR, interquartile range; SD, standard deviation

Patients with lower levels of albuminuria (UACR <200 mg/g) were taken primarily from the DECLARE_CKD_ data set with T2D patients only. We therefore estimated event rates and the dapagliflozin treatment effects for participants without T2D with a UACR <200 mg/g using a Poisson distribution because neither trial enrolled patients with low UACR but without T2D [26]. To do this, we applied adjustment factors for UACR and T2D status to the DECLARE_CKD_ data to estimate rates for patients without T2D with lower levels of albuminuria, relative to the treatment effect observed in patients with or without T2D in the DAPA-CKD trial (Supplementary data, Tables S1 and S2). The prevalence of T2D in the broad CKD population was assumed equivalent to the DAPA-CKD trial.

### Economic model

This analysis adapts a published Markov model for the DAPA-CKD trial population [27], which modelled disease progression by discrete eGFR-defined health states and health states for patients on kidney replacement therapy (KRT).

The primary outcome was the incremental cost-effectiveness ratio (ICER), expressed as the difference in costs per quality-adjusted life year (QALY) gained. Willingness-to-pay (WTP) thresholds specified for the presented analysis were $28 777/QALY, $39 063/QALY, $35 261/QALY and $53 671/QALY (£20 000/QALY, €30 000/QALY, €25 000/QALY and ¥5 million/QALY) for the UK, Spain, Italy and Japan, respectively.

**Figure 1:**
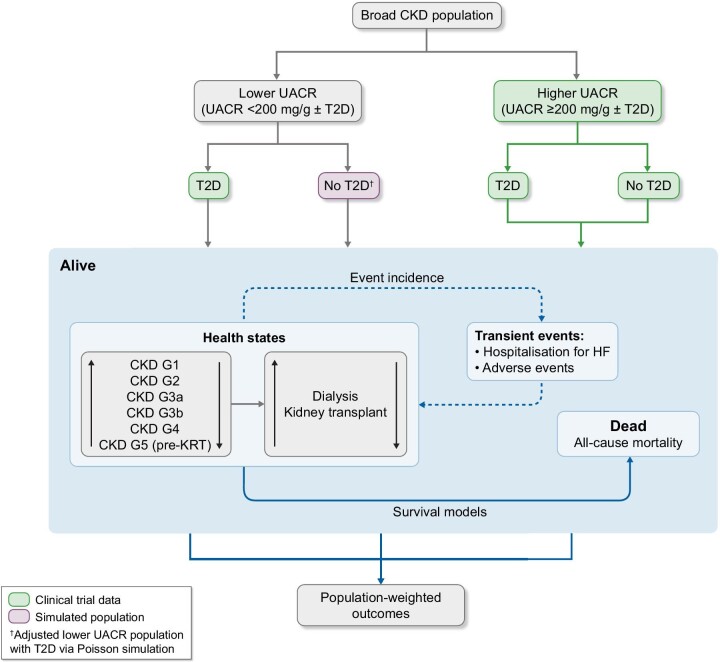
Model schematic for estimation of outcomes in the broad CKD population. HF: heart failure; UACR: urine albumin to creatinine ratio.

The adapted model estimated outcomes separately according to T2D status since the extrapolation estimates effects for patients without T2D and lower levels of albuminuria. The model outcomes are a weighted average according to a defined proportion of lower versus higher UACR (Fig. 1). The model assumes that 75% of the broad CKD population consists of patients with lower UACR (<200 mg/g) and 25% with higher UACR (≥200 mg/g), as observed in the DECLARE_CKD_ population, which had no albuminuria inclusion criterion.

The model assumes a constant rate of discontinuation, which was applied to all patients receiving treatment with dapagliflozin in each modelled cycle. Upon discontinuing treatment with dapagliflozin, patients were assumed to be treated with standard therapy only and subject to the risk of outcomes associated with that treatment arm.

### Disease progression

For patients with higher UACR, treatment dependent transition matrices were derived from the DAPA-CKD trial for the first 4 months of follow-up and from Month 4 thereafter, so as to replicate observed patterns of eGFR trajectory (see Supplementary data, Tables S3 and S4) [13]. For patients with lower UACR, transition matrices were derived by similarly using DECLARE_CKD_ data for CKD G1–G3 and DAPA-CKD for CKD G4 and G5, consistent with risk stratification according to guidelines (see Supplementary data, Tables S5 and S6) [28]. The model derives transitions for post-KRT outcomes from a systematic literature review of CKD modelling [29], as these events were rare in the DECLARE-TIMI 58 and DAPA-CKD trials.

To avoid double counting of mortality, we derived transition probabilities excluding the transition to death. We calculated the role of altered kidney function on the risk of death by parametric survival modelling, described below.

### Mortality and event incidence

We extrapolated the incidence of all-cause mortality using adjusted parametric survival equations, in accordance with established guidelines [30]. Health state defined by eGFR were time-updated to estimate the dynamic mortality risk associated with progression. Of the distributions evaluated, we considered the Weibull distribution to yield the most consistent fits by albuminuria stratification to the pooled trial data (Supplementary data, Fig. S1).

We used adjusted generalized estimating equations to capture the incidence of hospitalization for heart failure (HHF), including first and recurrent events. As with mortality, time-updated eGFR captured the effect of disease evolution on event risk. We conducted all analyses from an intention-to-treat perspective and validated against observed data (Supplementary data, Fig. S2).

Patients could experience treatment-related adverse events of interest at a constant rate, incurring management costs and utility decrements in the incident cycle only. Considered adverse events were volume depletion, major hypoglycaemic events, fractures, diabetic ketoacidosis and amputation.

### Resource use and costs

The analysis considered only direct costs from healthcare payer perspectives. We discounted costs at an annual rate of 3.5% in the UK, 3% in Spain and Italy and 2% in Japan, according to local guidelines [30–33]. Cost inputs specific to each country are presented in 2022 US dollars and native currencies in Supplementary data, Tables S7 and S8, respectively.

### Health-related quality of life

As EuroQol-5 Dimension 5 Levels (EQ-5D-5L) data were not recorded in the DECLARE-TIMI 58 trial, utility estimates were solely derived from patient-level EQ-5D-5L data from the DAPA-CKD trial (Table 2). Notably, utility values for each setting were derived using country-specific life tables and tariffs; further details are available in published material [14]. The model assumes that patients with lower UACR are adequately represented by HRQoL outcomes in patients with higher albuminuria; in effect, that patient utility is driven only by eGFR-defined stage and the incidence of complications, in line with other published CKD models [29]. We also discounted benefits annually at a rate of 3.5% in the UK, 3% in Spain and Italy, and 2% in Japan [30–33].

**Table 2:** Health state utility values and event-related disutility modifiers for the UK, Japan, Spain and Italy.

	Utility or utility decrement				
Parameter	UK	Italy	Spain	Japan	Source
Health-state utility					
CKD G1	0.77 (0.01)	0.85 (0.01)	0.83 (0.01)	0.80 (0.01)	DAPA-CKD [34]
CKD G2	0.77 (0.01)	0.85 (0.01)	0.83 (0.01)	0.80 (0.01)	DAPA-CKD [34]
CKD G3a	0.77 (0.01)	0.86 (0.01)	0.83 (0.01)	0.80 (0.01)	DAPA-CKD [34]
CKD G3b	0.77 (0.01)	0.86 (0.01)	0.84 (0.01)	0.80 (0.01)	DAPA-CKD [34]
CKD G4	0.76 (0.01)	0.85 (0.01)	0.84 (0.01)	0.79 (0.01)	DAPA-CKD [34]
CKD G5, pre-KRT	0.73 (0.01)	0.73 (0.01)	0.83 (0.01)	0.76 (0.01)	DAPA-CKD [34]
Dialysis	0.68 (0.01)	0.78 (0.01)	0.79 (0.01)	0.73 (0.01)	DAPA-CKD [34]
Transplant	0.71 (0.07)	0.71 (0.07)	0.77 (0.01)	0.74 (0.07)	DAPA-CKD [34]
Event-related disutility modifier					
HHF	–0.09 (0.04)	–0.08 (0.04)	–0.07 (0.03)	–0.06 (0.03)	DAPA-CKD [34]
Volume depletion	–0.05 (0.01)	–0.05 (0.01)	–0.05 (0.01)	–0.05 (0.01)	McEwan *et al*. [35]
Major hypoglycaemic events	–0.01 (0.00)	–0.01 (0.00)	–0.01 (0.00)	–0.01 (0.00)	Beaudet *et al*. [36]; Currie *et al*. [37]
Diabetic ketoacidosis	–0.01 (0.01)	–0.01 (0.01)	–0.01 (0.01)	–0.01 (0.01)	Peasgood *et al*. [38]
Fracture	–0.09 (0.03)	–0.05 (0.03)	–0.07 (0.03)	–0.05 (0.02)	DAPA-CKD [34]
Amputation	–0.26 (0.05)	–0.32 (0.05)	–0.26 (0.05)	–0.18 (0.04)	DAPA-CKD [34]

### Subgroup analyses

Key subgroups were established for the analysis to assess model outcomes stratified by T2D status (with and without) and level of albuminuria (lower: <200 mg/g; higher: ≥200 mg/g) to yield four subgroups for evaluation.

### Scenario analyses

The DAPA-CKD and DECLARE-TIMI 58 trials had median follow-up periods of 2.4 years and 4.2 years, respectively. Given that our model predicts outcomes over a lifetime horizon, we considered varying time horizons to characterize the evolution of the cost-effectiveness of dapagliflozin.

### Sensitivity analyses

We conducted one-way sensitivity analyses to demonstrate the influence of varying key parameter values on modelled outcomes. We conducted probabilistic sensitivity analysis to assess the influence of uncertainty across all model parameters on health economic outcomes. For each analysis, 1000 replicates were generated in which the parameters sampled included patient baseline demographic characteristics, direct costs, utilities, adverse event risks and treatment efficacy.

Probabilistic sensitivity analyses were also conducted separately for the assessed subgroups for each of the four countries. Specific to the extrapolative analysis in the population with low UACR without T2D, the parameters estimating HHF and mortality effects were also sampled. Results were evaluated on the cost-effectiveness plane and plotted with 95% credibility ellipses.

## RESULTS

### Base case analysis

In the broad CKD population, dapagliflozin is predicted to slow the rate of CKD progression and lower the incidence of HHF. Patients treated with dapagliflozin were likely to spend 1.08 more years in the earlier stages of CKD (stages G1 to G4) versus those treated with standard therapy alone (dapagliflozin 14.9 years; standard therapy 13.8 years; Table 3). Patients treated with dapagliflozin had a lower rate of HHF (119 versus 140 per 1000 patient-years).

**Table 3:** Base case clinical outcomes for the broad CKD population.

Outcome	Dapagliflozin plus standard therapy	Standard therapy	Incremental
Mean time in each CKD stage, years			
CKD G1	2.96	2.62	0.34
CKD G2	6.16	5.77	0.39
CKD G3	3.36	3.18	0.18
CKD G4	2.40	2.23	0.17
CKD G5 (pre-KRT)	1.28	1.30	–0.02
Dialysis	0.18	0.19	–0.01
Transplant	0.71	0.75	–0.03
Event incidence, per 1000 patients			
HHF	119	140	–21

Note that results are presented for the UK setting, there will be some variation in outcomes owing to the application of country-specific life tables.

These clinical benefits lead to extended life expectancy versus patients treated with standard therapy alone (Table 4), with mean predicted increase in life expectancy by 0.60 years in those treated with dapagliflozin (12.5 years) versus those treated with standard therapy alone (11.9 years) in the UK setting. Other settings demonstrated similar improvements in patient life expectancy (Spain 0.64; Italy 0.66 years; Japan 0.84).

**Table 4:** Base case health economic outcomes for the broad CKD population in the UK, Japan, Spain and Italy.

Outcome	Dapagliflozin plus standard therapy	Standard therapy	Incremental
UK			
Total costs	$63 243	$58 232	$5011
Drug acquisition	$6857	$854	$6002
CKD management (pre-KRT)	$28 690	$27 900	$790
KRT	$23 666	$25 362	–$1696
HHF	$642	$784	–$142
Adverse events	$3388	$3331	$57
Total LYs gained	12.51	11.91	0.60
Total QALYs gained	9.56	9.09	0.47
ICER (per LY gained)			$8293/LY
ICER (per QALY gained)			$10 676/QALY
Incremental NMB			$8496
Spain			
Total costs	$156 466	$148 659	$7807
Drug acquisition	$5679	$839	$4840
CKD management (pre-KRT)	$105 376	$100 171	$5205
KRT	$39 782	$42 546	–$2764
HHF	$475	$578	$103
Adverse events	$5154	$4524	$629
Total LYs gained	13.00	12.36	0.64
Total QALYs gained	10.75	10.21	0.54
ICER (per LY gained)			$12 124/LY
ICER (per QALY gained)			$14 479/QALY
Incremental NMB			$13 255
Italy			
Total costs	$67 999	$63 593	$4406
Drug acquisition	$6177	$1297	$4880
CKD management (pre-KRT)	$30 502	$29 175	$1327
KRT	$27 202	$29 014	–$1812
HHF	$567	$688	–$122
Adverse events	$3252	$3180	$72
Total LYs gained	13.09	12.43	0.66
Total QALYs gained	11.16	10.60	0.57
ICER (per LY gained)			$6701/LY
ICER (per QALY gained)			$7771/QALY
Incremental NMB			$15 587
Japan			
Total costs	$88 386	$79 115	$9271
Drug acquisition	$9749	$730	$9019
CKD management (pre-KRT)	$42 230	$40 486	$1744
KRT	$31 310	$33 037	–$1728
HHF	$1906	$2314	–$408
Adverse events	$3957	$3501	$456
Total LYs gained	14.75	13.91	0.84
Total QALYs gained	11.70	11.03	0.68
ICER (per LY gained)			$10 998/LY
ICER (per QALY gained)			$13 723/QALY
Incremental NMB			$26 989

LYs, life years; NMB, net monetary benefit.

Treatment with dapagliflozin in the broad CKD population led to mean lifetime QALY gains of 0.47, 0.54, 0.57 and 0.68 in the UK, Spain, Italy and Japan, respectively (Table 4). Differences between setting are borne from country specific utility tariffs, life tables and discounting rates.

Treatment with dapagliflozin led to increased overall costs versus placebo in the UK ($5011), Spain ($7807), Italy ($4406) and Japan ($9271). The main contributors of additional cost in those treated with dapagliflozin were additional drug acquisition costs and disease management costs resulting from extended life expectancy. These costs were partially offset by reductions in costs associated with KRT through delayed time to dialysis or transplant and a reduced rate of HHF over the lifetime model horizon.

Dapagliflozin was considered cost-effective with ICERs of $10 676/QALY, $14 479/QALY, $7771/QALY and $13 723/QALY in the UK, Spain, Italy and Japan, well below WTP thresholds ($28 777/QALY, $39 063/QALY, $35 261/QALY, $53 671/QALY, respectively) and estimated net monetary benefit of $8496, $13 255, $15 587 and $26 989 respectively. Health economic outcomes are presented in their native currency in Supplementary data, Table S9.

### Subgroup analysis

**Figure 2:**
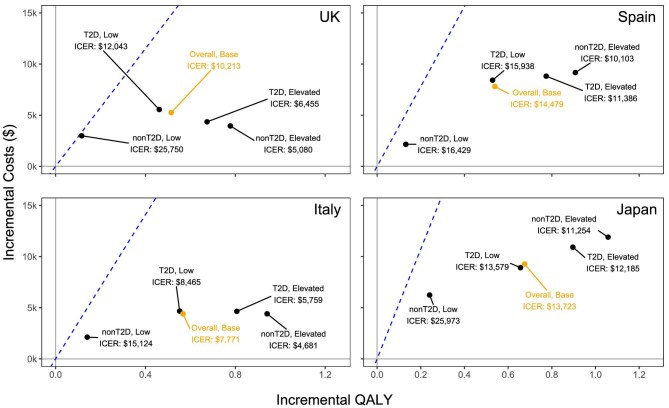
Subgroup analyses (top to bottom) in the UK, Spain, Italy and Japan dependent on UACR severity and T2D status. ‘Low’ and ‘elevated’ refer to albuminuria status (UACR <200 mg/g and UACR ≥200 mg/g respectively) while ‘Base’ refers to the base case analysis of 75% low and 25% elevated albuminuria. Dashed lines designate the WTP threshold of each country.

Across all subgroups analysed, dapagliflozin was considered cost effective at the corresponding WTP thresholds attributed to each country (Fig. 2). In all settings, the ICERs for patients with lower UACR and without T2D (UK $25 750; Spain $16 429; Italy $15 124; Japan $25 973) were highest, while ICERs for the subgroup with higher UACR and without T2D were lowest (UK $5080; Spain $10 103; Italy $4681; Japan $11 254). In all settings, a similar spread of incremental QALYs was observed; in terms of costs, T2D subgroups were associated with higher incremental costs than non-T2D subgroups and this differential was larger for the lower UACR than for the higher UACR groups.

### Scenario analysis

**Figure 3:**
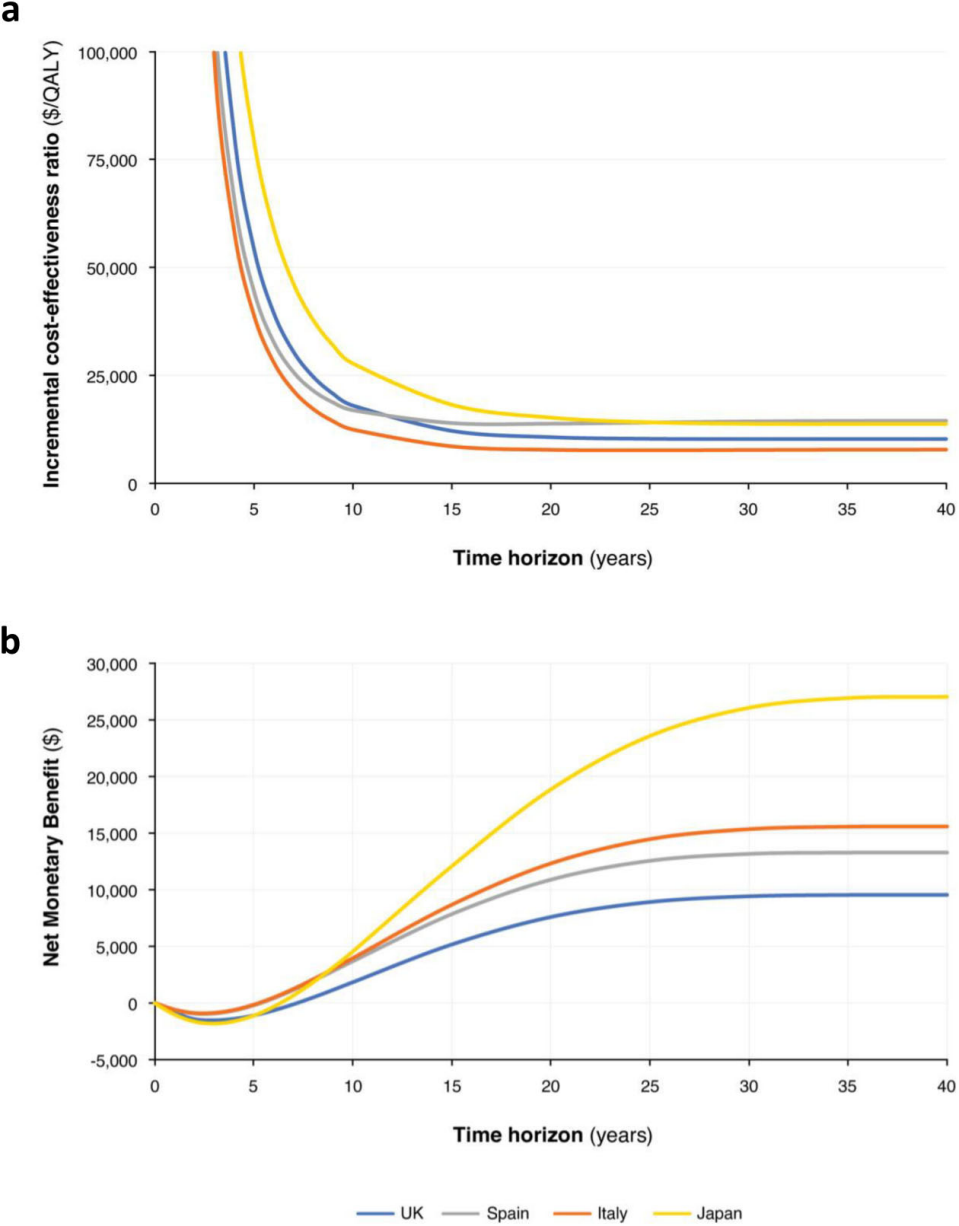
(**a**) ICER (US dollars per QALY) and (**b**) net monetary benefit evolution over time in the UK, Spain, Italy and Japan.

Treatment with dapagliflozin was estimated to lead to fewer patients progressing to ESKD and becoming hospitalized due to heart failure over a lifetime horizon. The largest changes in ICERs were observed during the early period, up to approximately 15 years, where increasing time horizons are associated with large decreases in the ICERs (Fig. 3). Beyond this point, the ICERs stabilize and show little change up to a lifetime time horizon. Similarly, the estimated net monetary benefit was negative over the initial years of treatment, before rising until 20 years from baseline, and the rate of increase levelled off across most settings by 25 years.

**Figure 4:**
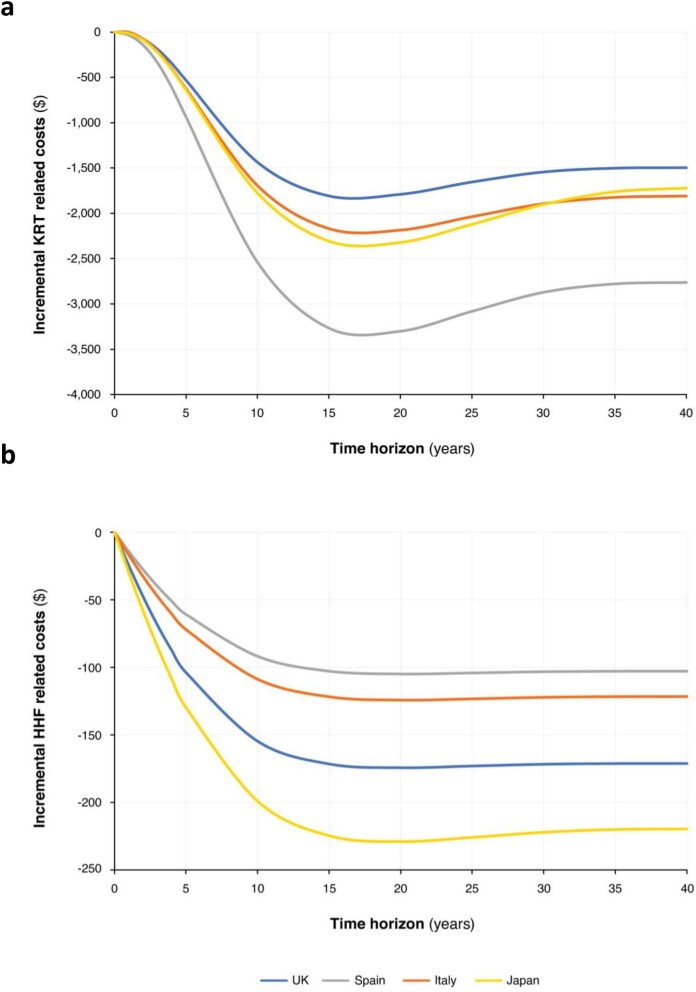
Incremental costs over time associated with (**a**) KRT and (**b**) HHF avoided.

Over the patient lifetime, treatment and disease management costs accrue, and these costs are offset in the dapagliflozin arm by reductions in HHF and KRT events (Fig. 4). While average, per-patient HHF cost reductions remain constant at longer time horizons, those related to avoided KRT exhibit a local minimum at around 15 years, followed by a slight increase before levelling off, likely due to a combined effect of survival and changes in disease progression as more patients transition to later stages of CKD.

### Sensitivity analysis

Deterministic sensitivity analyses revealed that, aside from discounting, subpopulation selection and time horizon had the greatest influence on outcomes. Analyses remained cost effective at the defined thresholds for each country for all parameter variations tested (Supplementary data, Table S10).

**Figure 5:**
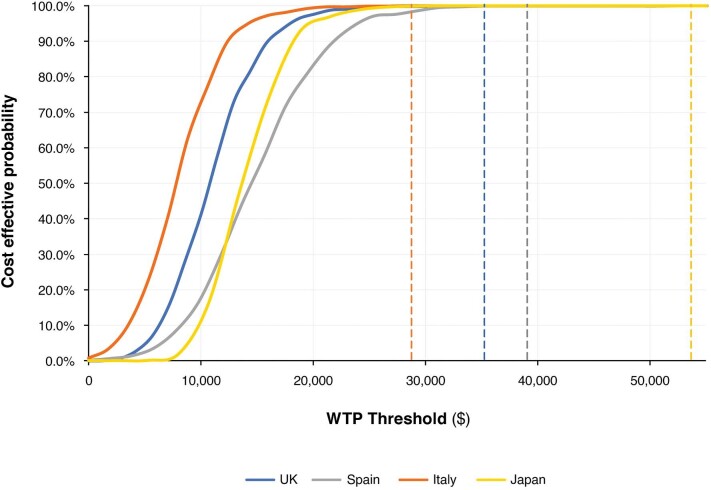
Cost-effectiveness acceptability curves for the UK, Spain, Italy and Japan. The probability of dapagliflozin in addition to standard therapy being cost-effective at the given WTP threshold versus standard therapy only. The specified WTP threshold for each country is denoted by the corresponding-coloured dotted line parallel to the y-axis.

Probabilistic sensitivity analysis showed that the model was robust to joint uncertainty of all parameters. In the UK, 99.8% of simulations were found to be cost effective at a threshold of $28 777/QALY (£20 000/QALY), 99.9% were cost effective at a threshold of $39 063 (€30 000/QALY) in Spain, whilst 100% were cost effective at thresholds of $35 261 (€25 000/QALY) in Italy and $53 671/QALY (¥5 000 000/QALY) in Japan (Fig. 5). Probabilistic sensitivity analyses across subgroups showed that cost-effectiveness outcomes were robust across simulations of the elevated UACR populations and low UACR population with T2D, while simulations in the population with low UACR without T2D showed less certainty in the cost-effectiveness outcomes for this population (see Supplementary data, Fig. S3).

## DISCUSSION

The present study sought to bridge a data gap to explore cost-effectiveness of dapagliflozin in patients with CKD including lower risk patients with lower levels of albuminuria with or without T2D. To model this broader population, individual patient data were pooled from randomized large outcome clinical trials of dapagliflozin plus standard therapy versus standard therapy alone that could inform overall survival and cardiorenal event risks. After adaptation and incorporation of these risk equations into a previously published cost-effectiveness model, results of the present study expand the evidence base of health economic outcomes in a population with CKD that is broader than the inclusion criteria of the DAPA-CKD trial. Analyses revealed ICERs below attributed WTP thresholds and positive net monetary benefit in all settings (UK, Spain, Italy and Japan), and outcomes were generally supportive of cost-effectiveness in patients with lower UACR (<200 mg/g) and without T2D. Outcomes were typically robust across deterministic and probabilistic sensitivity analyses.

Building on the previous cost-effectiveness analysis in the DAPA-CKD population [14], this analysis suggests that the clinical and economic value of dapagliflozin in comparison with usual care alone could be generalizable (albeit somewhat less pronounced) to patients with CKD who are not represented in the DECLARE-TIMI 58 or DAPA-CKD trials, including patients with albuminuria stage A2 without T2D. In line with the previous analysis, patients treated with dapagliflozin spent more time in CKD stages 1–4 (broad CKD population, 1.1 years; DAPA-CKD, 1.7 years) versus patients treated with standard therapy alone [14]. Moreover, dapagliflozin slowed the rate of HHF (broad CKD population, 21 events avoided; DAPA-CKD, 19 events avoided) and increased life expectancy (broad CKD population, 0.7 years; DAPA-CKD, 1.7 years) versus standard therapy alone. In comparison with the previous analysis in the higher UACR population only, potential benefits were lower as reflected by smaller incremental QALY and slightly smaller incremental costs in the context of the broader CKD population. The effect is expected since, while the rates of mortality, ESKD and cardiovascular events were lower relative to patients with more advanced eGFR and albuminuria stage of CKD, the presence of CKD will increase the risk of cardio-renal events and so the treatment effect of dapagliflozin was consistent [39]. Nevertheless, the analysis suggests that results would be considered cost effective at all country-specific thresholds for the broad CKD population, and even when restricted to the population with lower UACR. The substantial clinical benefits observed in the medium- to long-term suggest that, given management of CKD in patients within this broader population will typically lie with primary care physicians, earlier enrolment on reno-protective therapies such as dapagliflozin could be important in reducing the population level impact of CKD progression.

This study, as with any modelling analysis, is subject to several limitations. The most significant source of uncertainty relates to the requirement to extrapolate outcomes in patients without T2D and with lower UACR. Additionally, given the relative health of these patients, there are limited data available against which to externally validate these projections.

This analysis takes advantage of two trial data sets to generate a pooled population including patient-level data from 10 273 patients, including patients at lower risk of progression. Therefore, these patients would be expected to experience fewer events such as HHF or progression to advanced CKD, potentially confounding extrapolations for these individuals. The limitation is partly mitigated through the longer observation time for these patients (median trial follow-up was 4.2 years) and controlling for multiple variables including albuminuria and CKD state to exploit fully all available data to inform estimates.

The transition probabilities for post-KRT outcomes were estimated from a systematic review of published literature on modelling in CKD [29], given the small number of observed events in either trial.

Finally, the prevalence of albuminuria may vary between countries, and therefore country-specific prevalence data would best reflect outcomes specific to each setting. National prevalence data stratified by eGFR and albuminuria are limited and importantly, the DAPA-CKD trial threshold definition of higher albuminuria was 200 mg/g, a level that differs from KDIGO categories of albuminuria (A2, 30–300 mg/g; A3, >300 mg/g) [18, 24]. The relative proportions were instead approximated from the DECLARE_CKD_ trial data in which patients were not selected according to albuminuria status.

In conclusion, this analysis addresses a data gap that currently exists in patients with CKD. The country-specific results of the model suggest that dapagliflozin could be a cost-effective treatment option for the broader CKD population and irrespective of eGFR, UACR and T2D status in the UK, Spain, Italy and Japan.

## Supplementary Material

sfae025_Supplemental_File

## Data Availability

Data underlying the findings described in this manuscript may be obtained in accordance with AstraZeneca's data sharing policy described at: https://astrazenecagrouptrials.pharmacm.com/ST/Submission/Disclosure.
