# Supplementary material for: Dapagliflozin in chronic kidney disease: cost-effectiveness beyond the DAPA-CKD trial
Source: Clin Kidney J. 2024 Feb 9;17(2):sfae025. doi: 10.1093/ckj/sfae025 (PMC10883141; doi:10.1093/ckj/sfae025)
Supplement: sfae025_Supplemental_File [file sfae025_supplemental_file.docx]

**SUPPLEMENTARY MATERIALS**

**Title:** Dapagliflozin in chronic kidney disease: cost-effectiveness beyond the DAPA-CKD trial

**Authors:** Phil McEwan, PhD,^1^ Jason A. Davis, DPhil,^1^ Peter D. Gabb, BSc,^1^ David C. Wheeler, MD,^2^ Peter Rossing, MD DMSc,^3,4^ Glenn M. Chertow, MD-MPH,^5^ Ricardo Correa-Rotter, MD,^6^ Kouichi Tamura, MD PhD,^7^ Salvatore Barone, PharmD,^8^ Juan Jose Garcia Sanchez, MSc^9^

*(1) Health Economics and Outcomes Research Ltd, Cardiff, UK, (2) Department of Renal Medicine, University College London, London, UK, (3) Steno Diabetes Centre Copenhagen, Herlev Denmark, (4) Department of Clinical Medicine, University of Copenhagen, Copenhagen, Denmark, (5) Departments of Medicine and Epidemiology and Population Health, Stanford University School of Medicine, Stanford, CA, United States, (6) Department of Nephrology and Mineral Metabolism, National Medical Science and Nutrition Institute Salvador Zubiran, Mexico City, Mexico,* *(7) Department of Medical Science and Cardiorenal Medicine, Yokohama City University Graduate School of Medicine, Yokohama, Japan, (8) Global Medical Affairs, BioPharmaceuticals, AstraZeneca, Gaithersburg, Maryland, USA, (9) Global Market Access and Pricing, BioPharmaceuticals, AstraZeneca, Cambridge, UK*

**Corresponding author:** Phil McEwan

Email: [phil.mcewan@heor.co.uk](mailto:phil.mcewan@heor.co.uk)

# MODEL VALIDATION

Model predictions of survival were compared against the supporting trial data within the unified data set and in relation to the expert elicitation estimates of survival for CKD patients with elevated albuminuria (**Figure S1**). The estimates from the expert elicitation were in relation to patients with chronic kidney disease (CKD) and elevated albuminuria and therefore are indicative of patients modelled in **Figure S1**.

Weibull distributions were used for parametric survival models as they were most consistent with clinical expectations. As can be seen, the extrapolated placebo survival passes close to the mean estimated value. It should be noted that these estimates therefore do not apply for patients with low urine albumin-to-creatinine ratio (UACR), who had less severe CKD and lesser or no albuminuria. Nevertheless, these patients would be expected to have longer survival and, as shown, the extrapolations from trial data for the placebo curve come close to the upper limit of the 80% confidence interval of the elicitation estimates.

Additionally, the rate of hospitalisation for heart failure (HHF) was assessed in comparison with observed data from the unified data set (**Figure S2**), with stratification by low and elevated UACR. The model predictions were generally consistent with the observed rates of HHF in the DAPA-CKD and DECLARE TIMI 58 trials. Exceptions included the low UACR subgroup with eGFR < 45 ml/min per 1.73m^2^ at baseline, which deviated to a degree, likely due to lower event rates in the low UACR populations, which may increase the uncertainty.

# FIGURES

Figure S1. Validation plot of model survival versus trial Kaplan-Meier, Weibull distribution.

*Fully adjusted model predictions of overall survival using the Weibull distribution with trial Kaplan-Meier data for low (< 200 mg/g) and elevated (≥ 200 mg/g) UACR. Curves are overlaid with the 10-year expert elicitation estimation for survival in CKD patients with albuminuria without dapagliflozin (black, bounds refer to 80% confidence intervals). KM, Kaplan-Meier; UACR, urine albumin-to-creatinine ratio*


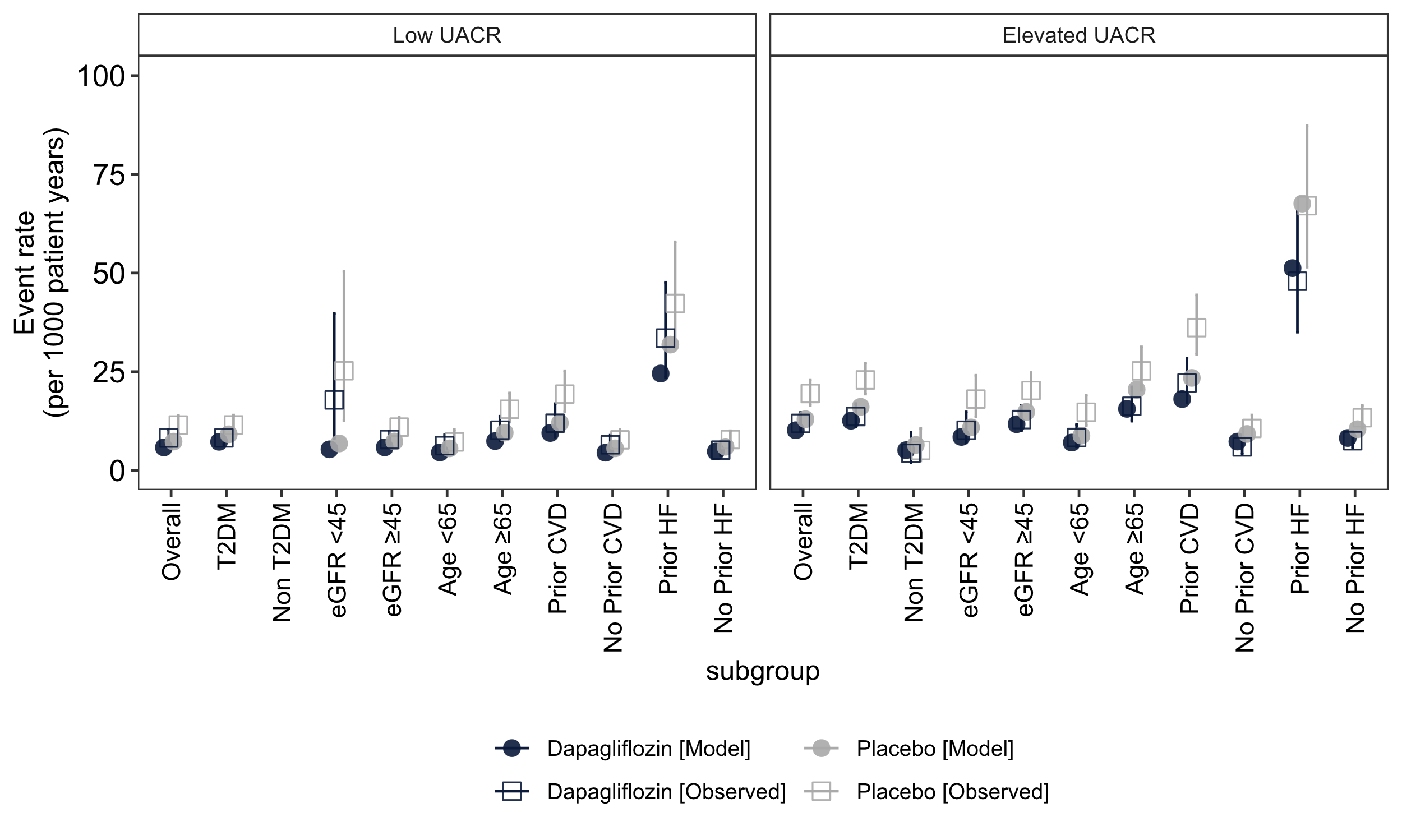
Figure S2. Validation plot of hospitalisation for heart failure event rate across subgroups.

*Fully adjusted model predictions of HHF event rate per 1,000 patient years are shown according to subgroup and stratified by low (< 200 mg/g) and elevated (≥ 200 mg/g) UACR populations. Data are not shown for the low UACR, non T2DM subgroup since there were too few patients (24 total from the DAPA-CKD trial) to inform an observed estimate against which to compare model results. CVD, cardiovascular disease; eGFR, estimated glomerular filtration rate; HF, heart failure; T2DM, type 2 diabetes mellitus; UACR, urine albumin creatinine ratio*

Figure S3.
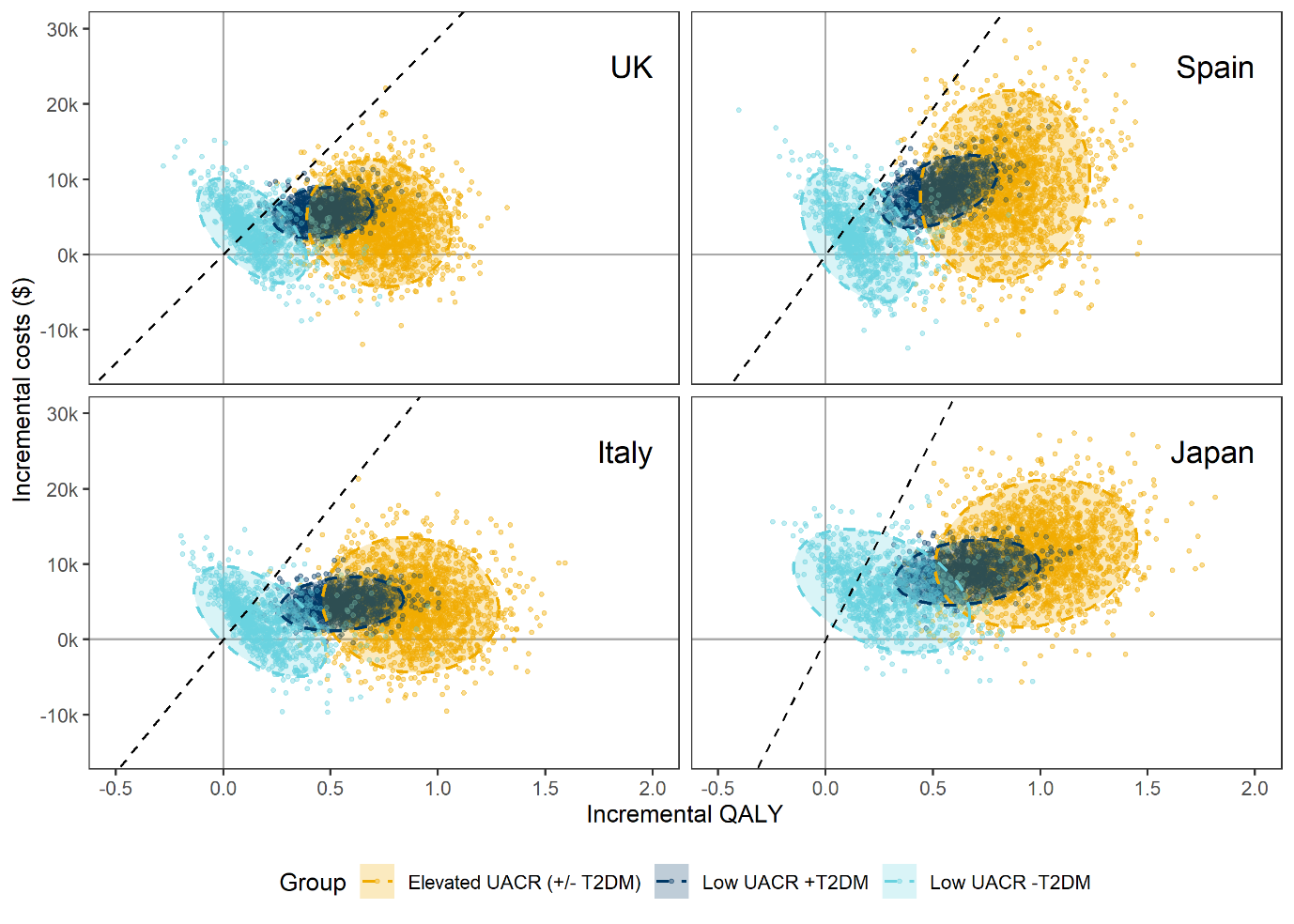
Probabilistic sensitivity analysis by country and subgroup (top to bottom) in the UK, Spain, Italy, and Japan dependent on UACR severity and T2D status.

*“Low” and “elevated” refer to albuminuria status (UACR < 200 mg/g and UACR ≥ 200 mg/g respectively). Dashed line indicates the WTP threshold of each country. QALY, quality adjusted life years; T2D, type 2 diabetes mellitus; UACR, urine albumin creatinine ratio*

# TABLES

Table S1. Event rates and rate ratio, dapagliflozin versus placebo at various levels of UACR

| Endpoint | UACR (mg/g) | Placebo (95% CI) | Dapagliflozin (95% CI) | Ratio (95% CI) |
| --- | --- | --- | --- | --- |
| Primary endpoint (≥ 50% sustained eGFR decline or incidence of ESKD) | 30 | 3.40 (3.11–3.72) | 2.28 (1.87–2.78) | 0.67 (0.56–0.80) |
|  | 300 | 3.87 (3.56–4.21) | 2.55 (2.11–3.07) | 0.66 (0.56–0.78) |
|  | 1000 | 5.44 (5.08–5.82) | 3.42 (2.91–4.03) | 0.63 (0.55–0.72) |
| ≥ 50% sustained eGFR decline | 30 | 1.87 (1.67–2.10) | 1.11 (0.85–1.44) | 0.59 (0.48–0.73) |
|  | 300 | 2.17 (1.95–2.41) | 1.26 (0.98–1.62) | 0.58 (0.4–0.70) |
|  | 1000 | 3.19 (2.92–3.48) | 1.77 (1.42–2.20) | 0.55 (0.47–0.65) |
| Incidence of ESKD | 30 | 1.37 (1.21–1.56) | 1.06 (0.81–1.38) | 0.77 (0.58–1.03) |
|  | 300 | 1.60 (1.42–1.81) | 1.20 (0.93–1.55) | 0.75 (0.58–0.98) |
|  | 1000 | 2.40 (2.17–2.65) | 1.69 (1.35–2.11) | 0.70 (0.57–0.87) |

*eGFR, estimated glomerular filtration rate; ESKD, end-stage renal disease; UACR, urine albumin creatinine ratio*

Table S2. Event rates and rate ratio, dapagliflozin versus placebo at various levels of UACR by T2D status

| Endpoint | Baseline status | UACR (mg/g) | Ratio (95% CI) |
| --- | --- | --- | --- |
| Primary endpoint (≥ 50% sustained eGFR decline or incidence of ESKD) | Non-T2D | 30 | 0.55 (0.39–0.79) |
|  |  | 300 | 0.55 (0.40–0.75) |
|  |  | 1 000 | 0.54 (0.42–0.68) |
|  | T2D | 30 | 0.69 (0.56–0.86) |
|  |  | 300 | 0.68 (0.56–0.83) |
|  |  | 1 000 | 0.65 (0.56–0.77) |
| ≥ 50% sustained eGFR decline | Non-T2D | 30 | 0.56 (0.36–0.86) |
|  |  | 300 | 0.55 (0.37–0.82) |
|  |  | 1 000 | 0.54 (0.40–0.73) |
|  | T2D | 30 | 0.57 (0.45–0.73) |
|  |  | 300 | 0.57 (0.45–0.71) |
|  |  | 1 000 | 0.54 (0.45–0.66) |
| Incidence of ESKD | Non-T2D | 30 | 0.70 (0.39–1.26) |
|  |  | 300 | 0.69 (0.41–1.16) |
|  |  | 1 000 | 0.66 (0.45–0.96) |
|  | T2D | 30 | 0.78 (0.55–1.11) |
|  |  | 300 | 0.76 (0.55–1.06) |
|  |  | 1 000 | 0.72 (0.55–0.94) |
| *eGFR, estimated glomerular filtration rate; ESKD, end-stage kidney disease; T2D, type 2 diabetes mellitus; UACR, urine albumin creatinine ratio* | | | |

***Table S3. CKD transition matrix - dapagliflozin and standard therapy – elevated UACR subgroups***

| **Mean (SE)** | | **To** | | | | | | | | **Reference** |
| --- | --- | --- | --- | --- | --- | --- | --- | --- | --- | --- |
|  |  | **CKD 1** | **CKD 2** | **CKD 3a** | **CKD 3b** | **CKD 4** | **CKD 5** | **Dialysis** | **Kidney transplant** |  |
| **Months 0-4** | | | | | | | | | | |
| **From** | **CKD 1** | 0.586 (0.076) | 0.219 (0.064) | 0.049 (0.033) | 0.049 (0.033) | 0.024 (0.024) | 0.024 (0.024) | 0.024 (0.024) | 0.025 (0.024) | DAPA-CKD^1^ |
|  | **CKD 2** | 0.018 (0.005) | 0.709 (0.016) | 0.246 (0.015) | 0.019 (0.005) | 0.003 (0.002) | 0.003 (0.002) | 0.001 (0.001) | 0.001 (0.001) |  |
|  | **CKD 3a** | 0.001 (0.001) | 0.079 (0.006) | 0.749 (0.009) | 0.162 (0.008) | 0.008 (0.002) | 0.000 (0.000) | 0.000 (0.000) | 0.000 (0.000) |  |
|  | **CKD 3b** | 0.001 (0.000) | 0.005 (0.001) | 0.079 (0.004) | 0.812 (0.006) | 0.102 (0.005) | 0.001 (0.000) | 0.000 (0.000) | 0.000 (0.000) |  |
|  | **CKD 4** | 0.001 (0.001) | 0.003 (0.001) | 0.006 (0.002) | 0.143 (0.008) | 0.843 (0.008) | 0.004 (0.001) | 0.001 (0.001) | 0.001 (0.000) |  |
|  | **CKD 5** | 0.063 (0.060) | 0.125 (0.080) | 0.062 (0.058) | 0.124 (0.080) | 0.375 (0.118) | 0.125 (0.080) | 0.063 (0.059) | 0.062 (0.059) |  |
|  | **Dialysis** | 0.000 (0.000) | 0.000 (0.000) | 0.000 (0.000) | 0.000 (0.000) | 0.000 (0.000) | 0.000 (0.000) | 0.995 (0.099) | 0.005 (0.001) | Sugrue et al.^2^ |
|  | **Kidney transplant** | 0.000 (0.000) | 0.000 (0.000) | 0.000 (0.000) | 0.000 (0.000) | 0.000 (0.000) | 0.000 (0.000) | 0.007 (0.001) | 0.993 (0.099) |  |
| **Months 5 and onwards** | | | | | | | | | | |
| **From** | **CKD 1** | 0.891 (0.017) | 0.070 (0.014) | 0.009 (0.005) | 0.015 (0.007) | 0.006 (0.004) | 0.003 (0.003) | 0.003 (0.003) | 0.003 (0.003) | DAPA-CKD^1^ |
|  | **CKD 2** | 0.005 (0.001) | 0.909 (0.004) | 0.078 (0.004) | 0.006 (0.001) | 0.002 (0.001) | 0.000 (0.000) | 0.000 (0.000) | 0.000 (0.000) |  |
|  | **CKD 3a** | 0.001 (0.000) | 0.025 (0.001) | 0.913 (0.003) | 0.059 (0.002) | 0.002 (0.000) | 0.000 (0.000) | 0.000 (0.000) | 0.000 (0.000) |  |
|  | **CKD 3b** | 0.000 (0.000) | 0.001 (0.000) | 0.025 (0.001) | 0.938 (0.002) | 0.035 (0.001) | 0.000 (0.000) | 0.000 (0.000) | 0.000 (0.000) |  |
|  | **CKD 4** | 0.000 (0.000) | 0.000 (0.000) | 0.001 (0.000) | 0.035 (0.002) | 0.952 (0.002) | 0.010 (0.001) | 0.001 (0.000) | 0.000 (0.000) |  |
|  | **CKD 5** | 0.001 (0.001) | 0.002 (0.001) | 0.002 (0.001) | 0.001 (0.001) | 0.027 (0.005) | 0.920 (0.008) | 0.045 (0.006) | 0.002 (0.001) |  |
|  | **Dialysis** | 0.000 (0.000) | 0.000 (0.000) | 0.000 (0.000) | 0.000 (0.000) | 0.000 (0.000) | 0.000 (0.000) | 0.995 (0.099) | 0.005 (0.001) | Sugrue et al.^2^ |
|  | **Kidney transplant** | 0.000 (0.000) | 0.000 (0.000) | 0.000 (0.000) | 0.000 (0.000) | 0.000 (0.000) | 0.000 (0.000) | 0.007 (0.001) | 0.993 (0.099) |  |
| *CKD: chronic kidney disease; SE: standard error*  ** Mean values from transition probabilities identified in the Sugrue et al.*  *All values are expressed a mean (SE), with mean values representing monthly transition probabilities. Standard errors represent the variability applied in the probabilistic sensitivity analysis.* | | | | | | | | | | |

***Table S4. CKD transition matrix - placebo and standard therapy – elevated UACR subgroups***

| **Mean (SE)** | | **To** | | | | | | | | **Reference** |
| --- | --- | --- | --- | --- | --- | --- | --- | --- | --- | --- |
|  |  | **CKD 1** | **CKD 2** | **CKD 3a** | **CKD 3b** | **CKD 4** | **CKD 5** | **Dialysis** | **Kidney transplant** |  |
| **Months 0-4** | | | | | | | | | | |
| **From** | **CKD 1** | 0.375 (0.084) | 0.313 (0.081) | 0.156 (0.064) | 0.031 (0.030) | 0.031 (0.030) | 0.031 (0.030) | 0.031 (0.030) | 0.031 (0.030) | DAPA-CKD^1^ |
|  | **CKD 2** | 0.009 (0.003) | 0.770 (0.014) | 0.195 (0.013) | 0.016 (0.004) | 0.004 (0.002) | 0.002 (0.002) | 0.002 (0.002) | 0.001 (0.001) |  |
|  | **CKD 3a** | 0.002 (0.001) | 0.070 (0.005) | 0.774 (0.009) | 0.149 (0.007) | 0.004 (0.001) | 0.000 (0.000) | 0.000 (0.000) | 0.000 (0.000) |  |
|  | **CKD 3b** | 0.002 (0.001) | 0.004 (0.001) | 0.084 (0.005) | 0.826 (0.006) | 0.082 (0.005) | 0.001 (0.001) | 0.001 (0.000) | 0.000 (0.000) |  |
|  | **CKD 4** | 0.001 (0.001) | 0.002 (0.001) | 0.005 (0.002) | 0.127 (0.008) | 0.856 (0.009) | 0.007 (0.002) | 0.001 (0.001) | 0.001 (0.001) |  |
|  | **CKD 5** | 0.043 (0.041) | 0.174 (0.077) | 0.043 (0.042) | 0.044 (0.042) | 0.175 (0.077) | 0.348 (0.097) | 0.130 (0.068) | 0.043 (0.041) |  |
|  | **Dialysis** | 0.000 (0.000) | 0.000 (0.000) | 0.000 (0.000) | 0.000 (0.000) | 0.000 (0.000) | 0.000 (0.000) | 0.995 (0.099) | 0.005 (0.001) | Sugrue et al.^2^ |
|  | **Kidney transplant** | 0.000 (0.000) | 0.000 (0.000) | 0.000 (0.000) | 0.000 (0.000) | 0.000 (0.000) | 0.000 (0.000) | 0.007 (0.001) | 0.993 (0.099) |  |
| **Months 5 and onwards** | | | | | | | | | | |
| **From** | **CKD 1** | 0.884 (0.020) | 0.075 (0.016) | 0.015 (0.007) | 0.011 (0.006) | 0.004 (0.004) | 0.004 (0.004) | 0.004 (0.004) | 0.004 (0.004) | DAPA-CKD^1^ |
|  | **CKD 2** | 0.004 (0.001) | 0.915 (0.004) | 0.072 (0.004) | 0.008 (0.001) | 0.002 (0.001) | 0.000 (0.000) | 0.000 (0.000) | 0.000 (0.000) |  |
|  | **CKD 3a** | 0.000 (0.000) | 0.023 (0.001) | 0.910 (0.003) | 0.064 (0.002) | 0.003 (0.001) | 0.000 (0.000) | 0.000 (0.000) | 0.000 (0.000) |  |
|  | **CKD 3b** | 0.000 (0.000) | 0.001 (0.000) | 0.026 (0.001) | 0.931 (0.002) | 0.041 (0.001) | 0.000 (0.000) | 0.001 (0.000) | 0.000 (0.000) |  |
|  | **CKD 4** | 0.000 (0.000) | 0.001 (0.000) | 0.001 (0.000) | 0.028 (0.001) | 0.954 (0.002) | 0.014 (0.001) | 0.002 (0.000) | 0.000 (0.000) |  |
|  | **CKD 5** | 0.001 (0.001) | 0.001 (0.001) | 0.001 (0.001) | 0.002 (0.001) | 0.038 (0.005) | 0.910 (0.008) | 0.044 (0.005) | 0.003 (0.002) |  |
|  | **Dialysis** | 0.000 (0.000) | 0.000 (0.000) | 0.000 (0.000) | 0.000 (0.000) | 0.000 (0.000) | 0.000 (0.000) | 0.995 (0.099) | 0.005 (0.001) | Sugrue et al.^2^ |
|  | **Kidney transplant** | 0.000 (0.000) | 0.000 (0.000) | 0.000 (0.000) | 0.000 (0.000) | 0.000 (0.000) | 0.000 (0.000) | 0.007 (0.001) | 0.993 (0.099) |  |
| *CKD: chronic kidney disease; SE: standard error*  ** Mean values from transition probabilities identified in the Sugrue et al.*  *All values are expressed a mean (SE), with mean values representing monthly transition probabilities. Standard errors represent the variability applied in the probabilistic sensitivity analysis.* | | | | | | | | | | |

***Table S5. CKD transition matrix - dapagliflozin and standard therapy – low UACR subgroups***

| **Mean (SE)** | | **To** | | | | | | | | **Reference** |
| --- | --- | --- | --- | --- | --- | --- | --- | --- | --- | --- |
|  |  | **CKD 1** | **CKD 2** | **CKD 3a** | **CKD 3b** | **CKD 4** | **CKD 5** | **Dialysis** | **Kidney transplant** |  |
| **Months 0-4** | | | | | | | | | | |
| **From** | CKD 1 | 0.998 (0.001) | 0.001 (0.000) | 0.000 (0.000) | 0.000 (0.000) | 0.000 (0.000) | 0.000 (0.000) | 0.000 (0.000) | 0.000 (0.000) | DECLARE_CKD_^3^ |
|  | CKD 2 | 0.000 (0.000) | 0.998 (0.001) | 0.001 (0.000) | 0.000 (0.000) | 0.000 (0.000) | 0.000 (0.000) | 0.000 (0.000) | 0.000 (0.000) |  |
|  | CKD 3a | 0.001 (0.001) | 0.001 (0.001) | 0.993 (0.002) | 0.002 (0.001) | 0.001 (0.001) | 0.001 (0.001) | 0.001 (0.001) | 0.001 (0.001) |  |
|  | CKD 3b | 0.004 (0.004) | 0.004 (0.004) | 0.012 (0.007) | 0.965 (0.012) | 0.004 (0.004) | 0.004 (0.004) | 0.004 (0.004) | 0.004 (0.004) |  |
|  | CKD 4 | 0.001 (0.001) | 0.003 (0.001) | 0.006 (0.002) | 0.143 (0.008) | 0.843 (0.008) | 0.004 (0.001) | 0.001 (0.001) | 0.001 (0.000) | DAPA-CKD^1^ |
|  | CKD 5 | 0.063 (0.060) | 0.125 (0.080) | 0.062 (0.058) | 0.124 (0.080) | 0.375 (0.118) | 0.125 (0.080) | 0.063 (0.059) | 0.062 (0.059) |  |
|  | Dialysis | 0.000 (0.000) | 0.000 (0.000) | 0.000 (0.000) | 0.000 (0.000) | 0.000 (0.000) | 0.000 (0.000) | 0.995 (0.100) | 0.005 (0.000) | Sugrue et al.^2^ |
|  | Kidney transplant | 0.000 (0.000) | 0.000 (0.000) | 0.000 (0.000) | 0.000 (0.000) | 0.000 (0.000) | 0.000 (0.000) | 0.007 (0.001) | 0.993 (0.099) |  |
| **Months 5 and onwards** | | | | | | | | | | |
| **From** | CKD 1 | 0.970 (0.001) | 0.029 (0.001) | 0.001 (0.000) | 0.000 (0.000) | 0.000 (0.000) | 0.000 (0.000) | 0.000 (0.000) | 0.000 (0.000) | DECLARE_CKD_^3^ |
|  | CKD 2 | 0.012 (0.001) | 0.971 (0.001) | 0.015 (0.001) | 0.001 (0.000) | 0.000 (0.000) | 0.000 (0.000) | 0.000 (0.000) | 0.000 (0.000) |  |
|  | CKD 3a | 0.001 (0.000) | 0.029 (0.001) | 0.950 (0.002) | 0.019 (0.001) | 0.000 (0.000) | 0.000 (0.000) | 0.000 (0.000) | 0.000 (0.000) |  |
|  | CKD 3b | 0.001 (0.001) | 0.007 (0.001) | 0.038 (0.003) | 0.947 (0.004) | 0.006 (0.001) | 0.001 (0.000) | 0.000 (0.000) | 0.000 (0.000) |  |
|  | CKD 4 | 0.000 (0.000) | 0.000 (0.000) | 0.001 (0.000) | 0.035 (0.002) | 0.952 (0.002) | 0.010 (0.001) | 0.001 (0.000) | 0.000 (0.000) | DAPA-CKD^1^ |
|  | CKD 5 | 0.001 (0.001) | 0.002 (0.001) | 0.002 (0.001) | 0.001 (0.001) | 0.027 (0.005) | 0.920 (0.008) | 0.045 (0.006) | 0.002 (0.001) |  |
|  | Dialysis | 0.000 (0.000) | 0.000 (0.000) | 0.000 (0.000) | 0.000 (0.000) | 0.000 (0.000) | 0.000 (0.000) | 0.995 (0.100) | 0.005 (0.000) | Sugrue et al.^2^ |
|  | Kidney transplant | 0.000 (0.000) | 0.000 (0.000) | 0.000 (0.000) | 0.000 (0.000) | 0.000 (0.000) | 0.000 (0.000) | 0.007 (0.001) | 0.993 (0.099) |  |
| *CKD: chronic kidney disease; SE: standard error*  ** Mean values from transition probabilities identified in the Sugrue et al.*  *All values are expressed a mean (SE), with mean values representing monthly transition probabilities. Standard errors represent the variability applied in the probabilistic sensitivity analysis.* | | | | | | | | | | |

***Table S6. CKD transition matrix - placebo and standard therapy – low UACR subgroups***

| **Mean (SE)** | | **To** | | | | | | | | **Reference** |
| --- | --- | --- | --- | --- | --- | --- | --- | --- | --- | --- |
|  |  | **CKD 1** | **CKD 2** | **CKD 3a** | **CKD 3b** | **CKD 4** | **CKD 5** | **Dialysis** | **Kidney transplant** |  |
| **Months 0-4** | | | | | | | | | | |
| **From** | CKD 1 | 0.997 (0.001) | 0.001 (0.001) | 0.000 (0.000) | 0.000 (0.000) | 0.000 (0.000) | 0.000 (0.000) | 0.000 (0.000) | 0.000 (0.000) | DECLARE_CKD_^3^ |
|  | CKD 2 | 0.000 (0.000) | 0.998 (0.001) | 0.001 (0.000) | 0.000 (0.000) | 0.000 (0.000) | 0.000 (0.000) | 0.000 (0.000) | 0.000 (0.000) |  |
|  | CKD 3a | 0.001 (0.001) | 0.002 (0.001) | 0.995 (0.002) | 0.001 (0.001) | 0.001 (0.001) | 0.001 (0.001) | 0.001 (0.001) | 0.001 (0.001) |  |
|  | CKD 3b | 0.004 (0.004) | 0.008 (0.005) | 0.008 (0.005) | 0.965 (0.011) | 0.004 (0.004) | 0.004 (0.004) | 0.004 (0.004) | 0.004 (0.004) |  |
|  | CKD 4 | 0.001 (0.001) | 0.002 (0.001) | 0.005 (0.002) | 0.127 (0.008) | 0.856 (0.009) | 0.007 (0.002) | 0.001 (0.001) | 0.001 (0.001) | DAPA-CKD^1^ |
|  | CKD 5 | 0.043 (0.041) | 0.174 (0.077) | 0.043 (0.042) | 0.044 (0.042) | 0.175 (0.077) | 0.348 (0.097) | 0.130 (0.068) | 0.043 (0.041) |  |
|  | Dialysis | 0.000 (0.000) | 0.000 (0.000) | 0.000 (0.000) | 0.000 (0.000) | 0.000 (0.000) | 0.000 (0.000) | 0.995 (0.100) | 0.005 (0.000) | Sugrue et al.^2^ |
|  | Kidney transplant | 0.000 (0.000) | 0.000 (0.000) | 0.000 (0.000) | 0.000 (0.000) | 0.000 (0.000) | 0.000 (0.000) | 0.007 (0.001) | 0.993 (0.099) |  |
| **Months 5 and onwards** | | | | | | | | | | |
| **From** | CKD 1 | 0.970 (0.001) | 0.029 (0.001) | 0.001 (0.000) | 0.000 (0.000) | 0.000 (0.000) | 0.000 (0.000) | 0.000 (0.000) | 0.000 (0.000) | DECLARE_CKD_^3^ |
|  | CKD 2 | 0.011 (0.001) | 0.969 (0.001) | 0.018 (0.001) | 0.002 (0.000) | 0.000 (0.000) | 0.000 (0.000) | 0.000 (0.000) | 0.000 (0.000) |  |
|  | CKD 3a | 0.001 (0.000) | 0.033 (0.001) | 0.942 (0.002) | 0.023 (0.001) | 0.001 (0.000) | 0.000 (0.000) | 0.000 (0.000) | 0.000 (0.000) |  |
|  | CKD 3b | 0.001 (0.001) | 0.007 (0.001) | 0.041 (0.003) | 0.941 (0.004) | 0.010 (0.001) | 0.000 (0.000) | 0.000 (0.000) | 0.000 (0.000) |  |
|  | CKD 4 | 0.000 (0.000) | 0.001 (0.000) | 0.001 (0.000) | 0.028 (0.001) | 0.954 (0.002) | 0.014 (0.001) | 0.002 (0.000) | 0.000 (0.000) | DAPA-CKD^1^ |
|  | CKD 5 | 0.001 (0.001) | 0.001 (0.001) | 0.001 (0.001) | 0.002 (0.001) | 0.038 (0.005) | 0.910 (0.008) | 0.044 (0.005) | 0.003 (0.002) |  |
|  | Dialysis | 0.000 (0.000) | 0.000 (0.000) | 0.000 (0.000) | 0.000 (0.000) | 0.000 (0.000) | 0.000 (0.000) | 0.995 (0.100) | 0.005 (0.000) | Sugrue et al.^2^ |
|  | Kidney transplant | 0.000 (0.000) | 0.000 (0.000) | 0.000 (0.000) | 0.000 (0.000) | 0.000 (0.000) | 0.000 (0.000) | 0.007 (0.001) | 0.993 (0.099) |  |
| *CKD: chronic kidney disease; SE: standard error*  ** Mean values from transition probabilities identified in the Sugrue et al.*  *All values are expressed a mean (SE), with mean values representing monthly transition probabilities. Standard errors represent the variability applied in the probabilistic sensitivity analysis.* | | | | | | | | | | |

Table S7. Cost inputs for the UK, Japan, Spain and Italy (costs in US dollars and inflated to 2022 values)

| **Parameter** | **UK** | **Spain** | **Italy** | **Japan** |
| --- | --- | --- | --- | --- |
| **Treatment (per annum)** | | | | |
| Dapagliflozin | 713 ^4^ | 594 ^5^ | 594 ^6^ | 1 038 ^7^ |
| Standard therapy | 72 ^4^ | 68 ^5^ | 104 ^8^ | 53 ^7^ |
| Treatment monitoring^a^ | 264 ^9^ | 60 ^10^ | 63 ^10^ | 65 ^10^ |
| **CKD management (per annum)** | | | | |
| CKD G1 | 1 841 ^11^ | 8 414 ^12^ | 1 606 ^13^ | 1 360 ^14^ |
| CKD G2 | 1 841 ^11^ | 7 656 ^12^ | 2 066 ^13^ | 1 666 ^14^ |
| CKD G3a | 1 841 ^11^ | 8 053 ^12^ | 2 170 ^13^ | 3 820 ^14^ |
| CKD G3b | 1 841 ^11^ | 8 053 ^12^ | 3 153 ^13^ | 3 820 ^14^ |
| CKD G4 | 6 445 ^11^ | 13 518 ^15^ | 5 330 ^13^ | 8 988 ^14^ |
| CKD G5, pre-KRT | 22 599 ^11^ | 22 915 ^15^ | 6 473 ^13^ | 11 198 ^14^ |
| Dialysis | 51 056 ^16^ | 82 206 ^15^ | 48 523 ^17^ | 52 096 ^18^ |
| Transplant | 30 694 ^19^ | 37 447 ^20^ | 102 944 ^21^ | 55 403 ^22^ |
| **Events** | | | | |
| HHF | 7 824 ^23^ | 5 550 ^20^ | 6 573 ^24^ | 11 628 ^25^ |
| Volume depletion | 48 ^26^ | 4 430 ^20^ | 63 ^10^ | 63 ^10^ |
| Major hypoglycaemic event | 547 ^27^ | 1 149 ^28^ | 3 517 ^29^ | 54 ^30^ |
| Diabetic ketoacidosis | 3 422 ^31^ | 6 614 ^20^ | 5 352 ^29^ | 9 119 ^32^ |
| Fracture | 3 792 ^33^ | 7 041 ^34^ | 3 379^b^ | 2 826 ^35^ |
| Amputation | 21 036 ^36^ | 14 983 ^20^ | 15 904^c^ | 9 958 ^37^ |
| *CKD, chronic kidney disease; HHF, hospitalisation for heart failure; KRT, kidney replacement therapy*  *^a^assumed to require one outpatient clinical/GP visit, for the first year of treatment only; ^b^DRG 250; ^c^DRG 113* | | | | |

Table S8. Cost inputs for the UK, Japan, Spain and Italy (native currency, inflated to 2022 values)

| **Parameter** | **UK** | **Spain** | **Italy** | **Japan** |
| --- | --- | --- | --- | --- |
| **Treatment (per annum)** | | | | |
| Dapagliflozin | £495^[4]^ | € 380^[5]^ | € 421^[6]^ | ¥96 689^[7]^ |
| Standard therapy | £50^[4]^ | € 43^[5]^ | € 74^[8]^ | ¥4 891^[7]^ |
| Treatment monitoring^a^ | £184^[9]^ | € 43^[10]^ | € 45^[10]^ | ¥5 869^[10]^ |
| **CKD management (per annum)** | | | | |
| CKD G1 | £1 279^[11]^ | € 5 385^[12]^ | € 1 139^[13]^ | ¥126 706^[14]^ |
| CKD G2 | £1 279^[11]^ | € 4 900^[12]^ | € 1 464^[13]^ | ¥155 215^[14]^ |
| CKD G3a | £1 279^[11]^ | € 5 154^[12]^ | € 1 538^[13]^ | ¥355 833^[14]^ |
| CKD G3b | £1 279^[11]^ | € 5 154^[12]^ | € 2 235^[13]^ | ¥355 833^[14]^ |
| CKD G4 | £4 479^[11]^ | € 8 651^[15]^ | € 3 778^[13]^ | ¥837 317^[14]^ |
| CKD G5, pre-KRT | £15 706^[11]^ | € 14 665^[15]^ | € 4 589^[13]^ | ¥1 043 215^[14]^ |
| Dialysis | £35 484^[16]^ | € 52 612^[15]^ | € 34 403^[17]^ | ¥4 853 219^[18]^ |
| Transplant | £21 332^[19]^ | € 23 966^[20]^ | € 72 987^[21]^ | ¥5 157 879^[22]^ |
| **Events** | | | | |
| HHF | £5 438^[23]^ | € 3 552^[20]^ | € 4 660^[24]^ | ¥1 083 253^[25]^ |
| Volume depletion | £34^[26]^ | € 2 834^[20]^ | € 45^[10]^ | ¥5 867^[10]^ |
| Major hypoglycaemic event | £380^[27]^ | € 735^[28]^ | € 2 493^[29]^ | ¥5 058^[30]^ |
| Diabetic ketoacidosis | £2 379^[31]^ | € 4 233^[20]^ | € 3 794^[29]^ | ¥849 519^[32]^ |
| Fracture | £2 636^[33]^ | € 4 506^[34]^ | € 2 396^b^ | ¥263 253^[35]^ |
| Amputation | £14 620^[36]^ | € 9 598^[20]^ | € 11 276^c^ | ¥927 658^[37]^ |
| *CKD: chronic kidney disease; HHF, hospitalisation for heart failure; KRT: kidney replacement therapy*  *^a^assumed to require one outpatient clinical/GP visit, for the first year of treatment only; ^b^DRG 250; ^c^DRG 113* | | | | |

Table S9. Base case health economic outcomes for the broad CKD population in the UK, Japan, Spain, and Italy (native currency)

| **Outcome** | **Dapagliflozin plus Standard Therapy** | **Standard Therapy** | **Incremental** |
| --- | --- | --- | --- |
| **UK** | | | |
| Total costs | £43,954 | £40,471 | £3,483 |
| *Drug acquisition* | £4,765 | £594 | £4,172 |
| *CKD management (pre-KRT)* | £19,940 | £19,391 | £549 |
| *KRT* | £16,448 | £17,627 | -£1,179 |
| *HHF* | £446 | £545 | -£99 |
| *Adverse events* | £2,355 | £2,315 | £40 |
| *Total LYs gained* | 12.51 | 11.91 | 0.60 |
| Total QALYs gained | 9.56 | 9.09 | 0.47 |
| ICER (per LY gained) | | | £5,764/LY |
| ICER (per QALY gained) | | | £7,421/QALY |
| Incremental NMB | | | £5,904 |
| **Spain** | | | |
| Total costs | € 100,146 | € 95,142 | € 5,004 |
| Drug acquisition | € 3,642 | € 537 | € 3,105 |
| CKD management (pre-KRT) | € 67,440 | € 64,109 | € 3,331 |
| KRT | € 25,461 | € 27,230 | -€ 1,769 |
| HHF | € 304 | € 370 | -€ 66 |
| Adverse events | € 3,298 | € 2,896 | € 403 |
| Total LYs gained | 13.00 | 12.36 | 0.64 |
| Total QALYs gained | 10.75 | 10.21 | 0.54 |
| ICER (per LY gained) | | | € 7,771/LY |
| ICER (per QALY gained) | | | € 9,281/QALY |
| Incremental NMB | | | € 11,171 |
| **Italy** | | | |
| Total costs | € 48,218 | € 45,093 | € 3,125 |
| Drug acquisition | € 4,386 | € 926 | € 3,461 |
| CKD management (pre-KRT) | € 21,626 | € 20,685 | € 941 |
| KRT | €19,286 | €20,571 | -€1,285 |
| HHF | € 402 | € 488 | -€ 86 |
| Adverse events | € 2,518 | € 2,424 | € 95 |
| Total LYs gained | 13.09 | 12.43 | 0.66 |
| Total QALYs gained | 11.16 | 10.60 | 0.57 |
| ICER (per LY gained) | | | € 4,753/LY |
| ICER (per QALY gained) | | | € 5,508/QALY |
| Incremental NMB | | | € 11,059 |
| **Japan** | | | |
| Total costs | ¥8,233,524 | ¥7,370,164 | ¥863,360 |
| Drug acquisition | ¥907,867 | ¥68,029 | ¥839,839 |
| CKD management (pre-KRT) | ¥3,934,126 | ¥3,771,701 | ¥162,425 |
| KRT | ¥2,916,652.20 | ¥3,077,578.79 | -¥160,926.59 |
| HHF | ¥106,267 | ¥126,728 | -¥20,461 |
| Adverse events | ¥368,611 | ¥326,128 | ¥42,483 |
| Total LYs gained | 14.75 | 13.91 | 0.84 |
| Total QALYs gained | 11.70 | 11.03 | 0.68 |
| ICER (per LY gained) | | | ¥1,024,169/LY |
| ICER (per QALY gained) | | | ¥1,277,919/QALY |
| Incremental NMB | | | ¥2,514,631 |
| *CKD: chronic kidney disease; HHF, Hospitalisation for heart failure; ICER: incremental cost-effectiveness ratio; KRT: kidney replacement therapy; QALY: quality-adjusted life year* | | | |

Table S10. Deterministic sensitivity analyses (costs in US dollars)

| **Scenario** | **UK** | | | | **Spain** | | | | **Italy** | | | | **Japan** | | | |
| --- | --- | --- | --- | --- | --- | --- | --- | --- | --- | --- | --- | --- | --- | --- | --- | --- |
|  | **Cost, $** | **QALYs** | **LYs** | **Cost/**  **QALY** | **Cost, $** | **QALYs** | **LYs** | **Cost/**  **QALY** | **Cost, $** | **QALYs** | **LYs** | **Cost/**  **QALY** | **Cost, $** | **QALYs** | **LYs** | **Cost/**  **QALY** |
| **Base case** | **5,011** | **0.47** | **0.60** | **10,676** | **7,807** | **0.54** | **0.64** | **14,479** | **4,406** | **0.57** | **0.66** | **7,771** | **9,271** | **0.68** | **0.84** | **13,723** |
| Model time horizon (10 years) | 2,885 | 0.15 | 0.19 | 19,220 | 2,807 | 0.17 | 0.20 | 16,930 | 2,149 | 0.17 | 0.20 | 12,419 | 4,847 | 0.18 | 0.22 | 27,667 |
| Model time horizon (Lifetime) | 5,011 | 0.47 | 0.60 | 10,676 | 7,807 | 0.54 | 0.64 | 14,479 | 4,406 | 0.57 | 0.66 | 7,771 | 9,271 | 0.68 | 0.84 | 13,723 |
| Cost discounting (0.00%) | 7,228 | 0.47 | 0.60 | 15,399 | 11,791 | 0.54 | 0.64 | 21,869 | 6,320 | 0.57 | 0.66 | 11,147 | 11,640 | 0.68 | 0.84 | 17,230 |
| Cost discounting (6.00%) | 4,121 | 0.47 | 0.60 | 8,781 | 5,566 | 0.54 | 0.64 | 10,323 | 3,342 | 0.57 | 0.66 | 5,893 | 6,531 | 0.68 | 0.84 | 9,667 |
| Benefit discounting (0.00%) | 5,011 | 0.77 | 0.99 | 6,527 | 7,807 | 0.82 | 0.99 | 9,483 | 4,406 | 0.87 | 1.01 | 5,066 | 9,271 | 0.92 | 1.15 | 10,082 |
| Benefit discounting (6.00%) | 5,011 | 0.35 | 0.44 | 14,532 | 7,807 | 0.37 | 0.44 | 21,018 | 4,406 | 0.39 | 0.45 | 11,316 | 9,271 | 0.39 | 0.49 | 23,521 |
| Adverse events (excluded) | 4,954 | 0.47 | 0.60 | 10,541 | 7,178 | 0.54 | 0.64 | 13,308 | 4,273 | 0.57 | 0.66 | 7,529 | 8,815 | 0.68 | 0.84 | 13,043 |
| Adverse events (included) | 5,011 | 0.47 | 0.60 | 10,676 | 7,807 | 0.54 | 0.64 | 14,479 | 4,406 | 0.57 | 0.66 | 7,771 | 9,271 | 0.68 | 0.84 | 13,723 |
| Health state costs (80% of mean) | 5,192 | 0.47 | 0.60 | 11,062 | 7,319 | 0.54 | 0.64 | 13,574 | 4,503 | 0.57 | 0.66 | 7,943 | 9,268 | 0.68 | 0.84 | 13,718 |
| Health state costs (120% of mean) | 4,830 | 0.47 | 0.60 | 10,290 | 8,295 | 0.54 | 0.64 | 15,385 | 4,309 | 0.57 | 0.66 | 7,600 | 9,274 | 0.68 | 0.84 | 13,728 |
| Event costs (80% of mean) | 5,039 | 0.47 | 0.60 | 10,737 | 7,828 | 0.54 | 0.64 | 14,517 | 4,431 | 0.57 | 0.66 | 7,814 | 9,315 | 0.68 | 0.84 | 13,788 |
| Event costs (120% of mean) | 4,982 | 0.47 | 0.60 | 10,615 | 7,786 | 0.54 | 0.64 | 14,441 | 4,382 | 0.57 | 0.66 | 7,729 | 9,227 | 0.68 | 0.84 | 13,658 |
| Adverse event costs (80% of mean) | 4,999 | 0.47 | 0.60 | 10,652 | 7,681 | 0.54 | 0.64 | 14,246 | 4,380 | 0.57 | 0.66 | 7,724 | 9,180 | 0.68 | 0.84 | 13,588 |
| Adverse event costs (120% of mean) | 5,022 | 0.47 | 0.60 | 10,700 | 7,933 | 0.54 | 0.64 | 14,713 | 4,433 | 0.57 | 0.66 | 7,819 | 9,362 | 0.68 | 0.84 | 13,858 |
| Intervention costs (80% of mean) | 3,919 | 0.47 | 0.60 | 8,350 | 6,871 | 0.54 | 0.64 | 12,743 | 3,468 | 0.57 | 0.66 | 6,117 | 7,501 | 0.68 | 0.84 | 11,103 |
| Intervention costs (120% of mean) | 6,103 | 0.47 | 0.60 | 13,002 | 8,743 | 0.54 | 0.64 | 16,216 | 5,345 | 0.57 | 0.66 | 9,426 | 11,041 | 0.68 | 0.84 | 16,343 |
| Comparator costs (80% of mean) | 5,002 | 0.47 | 0.60 | 10,658 | 7,798 | 0.54 | 0.64 | 14,463 | 4,393 | 0.57 | 0.66 | 7,747 | 9,262 | 0.68 | 0.84 | 13,710 |
| Comparator costs (120% of mean) | 5,020 | 0.47 | 0.60 | 10,694 | 7,816 | 0.54 | 0.64 | 14,496 | 4,420 | 0.57 | 0.66 | 7,796 | 9,280 | 0.68 | 0.84 | 13,736 |
| Health state utility (80% of mean) | 5,011 | 0.38 | 0.60 | 13,348 | 7,807 | 0.43 | 0.64 | 18,100 | 4,406 | 0.45 | 0.66 | 9,716 | 9,271 | 0.54 | 0.84 | 17,155 |
| Health state utility (120% of mean) | 5,011 | 0.56 | 0.60 | 8,895 | 7,807 | 0.65 | 0.64 | 12,083 | 4,406 | 0.66 | 0.66 | 6,678 | 9,271 | 0.81 | 0.84 | 11,435 |
| Event disutility (80% of mean) | 5,011 | 0.47 | 0.60 | 10,677 | 7,807 | 0.54 | 0.64 | 14,480 | 4,406 | 0.57 | 0.66 | 7,772 | 9,271 | 0.68 | 0.84 | 13,723 |
| Event disutility (120% of mean) | 5,011 | 0.47 | 0.60 | 10,675 | 7,807 | 0.54 | 0.64 | 14,479 | 4,406 | 0.57 | 0.66 | 7,771 | 9,271 | 0.68 | 0.84 | 13,723 |
| Adverse event disutility (80% of mean) | 5,011 | 0.47 | 0.60 | 10,673 | 7,807 | 0.54 | 0.64 | 14,478 | 4,406 | 0.57 | 0.66 | 7,770 | 9,271 | 0.68 | 0.84 | 13,722 |
| Adverse event disutility (120% of mean) | 5,011 | 0.47 | 0.60 | 10,679 | 7,807 | 0.54 | 0.64 | 14,480 | 4,406 | 0.57 | 0.66 | 7,773 | 9,271 | 0.68 | 0.84 | 13,724 |
| Discontinuation (0.00%) | 5,011 | 0.47 | 0.60 | 10,676 | 7,807 | 0.54 | 0.64 | 14,479 | 4,406 | 0.57 | 0.66 | 7,771 | 9,271 | 0.68 | 0.84 | 13,723 |
| Discontinuation (10.00%) | 5,011 | 0.47 | 0.60 | 10,676 | 7,807 | 0.54 | 0.64 | 14,479 | 4,406 | 0.57 | 0.66 | 7,771 | 9,271 | 0.68 | 0.84 | 13,723 |
| *eGFR, estimated glomerular filtration rate; LY, life year; QAL,; quality-adjusted life year; T2DM, type 2 diabetes mellitus* | | | | | | | | | | | | | | | | |

# REFERENCES

1. AstraZeneca Data on File. Clinical Study Report: A Study to Evaluate the Effect of Dapagliflozin on Renal Outcomes and Cardiovascular Mortality in Patients with Chronic Kidney Disease. 2020.

2. Sugrue DM WT, Rai S, et al, . Economic Modelling of Chronic Kidney Disease: A Systematic Literature Review to Inform Conceptual Model Design. Pharmacoeconomics. 37(12):1451-68. 2019.

3. Wiviott SD, Raz I, Bonaca MP, et al. Dapagliflozin and Cardiovascular Outcomes in Type 2 Diabetes. N Engl J Med. 2019;380(4):347-57.

4. Haymarket Media Group. Database of Prescription and Generic Drugs, Clinical Guidelines. Monthly Index of Medical Specialities. 2020. Available at: <https://www.mims.co.uk/> [Accessed 28/10/2020].

5. Portal Farma. Bot PLUS. 2022. Available at: <https://botplusweb.portalfarma.com/> [Accessed 17/11/2022].

6. Gazzetta Ufficiale Della Repubblica Italiana. Serie Generale n.73 del 28-03-2015. 2015. Available at: <https://www.gazzettaufficiale.it/eli/gu/2015/03/28/73/sg/pdf> [Accessed 13/12/2022].

7. Ministry of Health Labor and Welfare. List of NHI drug price standards and information on generic drugs. 2022. Available at: <https://www.mhlw.go.jp/topics/2022/04/tp20220401-01.html> [Accessed 14/02/2023].

8. Agenzia Italiana del Farmaco. Lista farmaci equivalenti Principio Attivo 17.05.2021. 2021. Available at: <https://www.aifa.gov.it/documents/20142/1504839/Lista_farmaci_equivalenti_Principio_Attivo_17.05.2021.pdf> [Accessed 13/12/2022].

9. Department of Health. NHS reference costs 2020 to 2021. 2022. Available at: <https://www.england.nhs.uk/costing-in-the-nhs/national-cost-collection/> [Accessed 16/11/2022].

10. World Health Organization. CHOosing Interventions that are Cost Effective (WHO-CHOICE). 2005. Available at: <https://www.who.int/teams/health-systems-governance-and-financing/economic-analysis/costing-and-technical-efficiency/quantities-and-unit-prices-(cost-inputs>) [Accessed 28/10/2020].

11. Kent S, Schlackow I, Lozano-Kühne J, et al. What is the impact of chronic kidney disease stage and cardiovascular disease on the annual cost of hospital care in moderate-to-severe kidney disease? BMC Nephrology. 2015;16(1):65.

12. Darbà J, Marsà A. Chronic kidney disease in Spain: analysis of patient characteristics, incidence and direct medical costs (2011-2017). J Med Econ. 2020;23(12):1623-9.

13. Jommi C, Armeni P, Battista M, et al. The Cost of Patients with Chronic Kidney Failure Before Dialysis: Results from the IRIDE Observational Study. Pharmacoecon Open. 2018;2(4):459-67.

14. Kondo M, Yamagata K, Hoshi SL, et al. Cost-effectiveness of chronic kidney disease mass screening test in Japan. Clin Exp Nephrol. 2012;16(2):279-91.

15. Lorenzo-Sellares V, Pedrosa MI, Santana-Expósito B, et al. Análisis de costes y perfil sociocultural del enfermo renal. Impacto de la modalidad de tratamiento. Nefrología. 2014;34(4):458-68.

16. National Institute for Health and Care Excellence. Renal replacement therapy and conservative management. NICE guideline [NG107]. 2018. Available at: <https://www.nice.org.uk/guidance/ng107> [Accessed 28/10/2020].

17. Vaccaro CM, Sopranzi F. A comparison between the costs of dialysis treatments in Marche Region, Italy: Macerata and Tolentino hospitals. Annali dell'Istituto Superiore di Sanità. 2017;53(4):344-9.

18. Takura T, Nakanishi T, Kawanishi H, et al. Cost-Effectiveness of Maintenance Hemodialysis in Japan. Therapeutic Apheresis and Dialysis. 2015;19(5):441-9.

19. NHS Blood and Transplant. Cost-effectiveness of transplantation. 2009. Available at: <https://nhsbtmediaservices.blob.core.windows.net/organ-donation-assets/pdfs/Organ_Donation_Registry_Fact_Sheet_7_21337.pdf> [Accessed 16/11/2022].

20. Región de Murcia Consejería de Salud. El Conjunto Mínimo Básico de Datos. Informe regional CMBD 2015 Urología. 2016. Available at: <https://www.murciasalud.es/pagina.php?id=154065> [Accessed 30/10/2020].

21. Cavallo MC, Sepe V, Conte F, et al. Cost-Effectiveness of Kidney Transplantation From DCD in Italy. Transplantation Proceedings. 2014;46(10):3289-96.

22. Kitazawa T, Matsumoto K, Fujita S, et al. Cost Analysis of Transplantation in Japan, Performed With the Use of the National Database. Transplant Proc. 2017;49(1):4-9.

23. Kent S, Briggs A, Eckermann S, et al. Are value of information methods ready for prime time? An application to alternative treatment strategies for NSTEMI patients. Int J Technol Assess Health Care. 2013;29(4):435-42.

24. Maggioni AP, Orso F, Calabria S, et al. The real-world evidence of heart failure: findings from 41 413 patients of the ARNO database. Eur J Heart Fail. 2016;18(4):402-10.

25. Kaku K, Haneda M, Sakamaki H, et al. Cost-effectiveness Analysis of Empagliflozin in Japan Based on Results From the Asian subpopulation in the EMPA-REG OUTCOME Trial. Clin Ther. 2019;41(10):2021-40.e11.

26. Personal Social Services Research Unit. Unit Costs of Health and Social Care. 2020. Available at: <https://www.pssru.ac.uk/project-pages/unit-costs/unit-costs-2020/> [Accessed 28/10/2020].

27. Hammer M, Lammert M, Mejías SM, et al. Costs of managing severe hypoglycaemia in three European countries. Journal of Medical Economics. 2009;12(4):281-90.

28. Parekh W, Hoskins N, Baker-Knight J, et al. The Economic Burden of Insulin-Related Hypoglycemia in Spain. Diabetes Therapy. 2017;8(4):899-913.

29. Nicolucci A. L’ipoglicemia: le varie dimensioni del problema in Italia. G AMD. 2014;17(suppl 3):5-9.

30. Ikeda Y, Kubo T, Oda E, et al. Retrospective analysis of medical costs and resource utilization for severe hypoglycemic events in patients with type 2 diabetes in Japan. J Diabetes Investig. 2019;10(3):857-65.

31. Dhatariya KK, Skedgel C, Fordham R. The cost of treating diabetic ketoacidosis in the UK: a national survey of hospital resource use. Diabetic Medicine. 2017;34(10):1361-6.

32. Lkhagva D, Kuwabara K, Matsuda S, et al. Assessing the impact of diabetes-related comorbidities and care on the hospitalization costs for patients with diabetes mellitus in Japan. J Diabetes Complications. 2012;26(2):129-36.

33. Department of Health. NHS reference costs 2019 to 2020. 2020. Available at: <https://www.england.nhs.uk/national-cost-collection/> [Accessed 28/10/2020].

34. Oblikue Consulting. eSalud - Información económica del sector sanitario. Available at: <http://esalud.oblikue.com/> [Accessed 28/10/2020].

35. Taguchi Y, Inoue Y, Kido T, et al. Treatment costs and cost drivers among osteoporotic fracture patients in Japan: a retrospective database analysis. Arch Osteoporos. 2018;13(1):45.

36. Alva ML, Gray A, Mihaylova B, et al. The impact of diabetes-related complications on healthcare costs: new results from the UKPDS (UKPDS 84). Diabetic Medicine. 2015;32(4):459-66.

37. Davis KL, Meyers J, Zhao Z, et al. High-Risk Atherosclerotic Cardiovascular Disease in a Real-World Employed Japanese Population: Prevalence, Cardiovascular Event Rates, and Costs. J Atheroscler Thromb. 2015;22(12):1287-304.
